# A study on the impact of DRG payment on physicians’ prescribing behavior in China: a case study of a healthcare consortium in J City

**DOI:** 10.3389/fpubh.2025.1532622

**Published:** 2025-04-28

**Authors:** Zhenguo Wang, Li Li, Yuwen Cui

**Affiliations:** ^1^Nanshan People's Hospital, Jinan, Shandong Province, China; ^2^School of Public Health, Shandong University, Jinan, Shandong Province, China

**Keywords:** DRG payment, physicians’ prescribing behavior, pharmaceutical costs, healthcare consortium, acute ischemic stroke

## Abstract

As one of the effective strategies adopted by countries worldwide to mitigate the rapid growth of healthcare expenditures, the DRG (Diagnosis-Related Group) payment system has been implemented in developed Western countries for both outpatient and inpatient care, focusing on costs associated with medical technologies, ancillary services, nursing care, and other healthcare-related expenses. However, in China, the excessive rise in consumable medical costs, particularly for pharmaceuticals, remains a primary driver of unreasonable healthcare expenditure growth. This study takes a healthcare alliance in City J, China, encompassing primary, secondary, and tertiary public hospitals, as a case example. By combining qualitative interviews with quantitative research methods, it explores the impact of DRG payment on physician prescribing behaviors. The results indicate that the DRG payment system significantly influences physician prescribing practices, a finding that holds after a series of robustness checks. Moreover, the effect varies across hospitals of different levels, with the DRG payment system having a more pronounced impact on pharmaceutical costs for acute ischemic stroke cases with two or fewer comorbidities. Therefore, the DRG payment system holds significant implications for the rational allocation of healthcare resources in China.

## 1 Introduction

In the current institutional environment of China, public hospitals hold a central position within the entire healthcare service system. However, with the continuous advancement of China’s market-oriented economic reforms, the public welfare nature of these hospitals has gradually diminished (1). The growth of healthcare costs has significantly outpaced the growth of GDP during the same period, posing a severe challenge to the public’s health security. To safeguard and improve the population’s health, many countries and regions have adopted healthcare policies centered around hospitals as the primary institutions (2). Establishing an effective medical insurance payment system to promote the reform of public hospitals has become a crucial approach for China in advancing high-quality, coordinated development in healthcare security and medical services. The Diagnosis-Related Group (DRG) payment system originated in the United States and has now been widely adopted in developed nations as well as some middle-and low-income countries (3, 4). As a refined hospital management tool, the DRG payment system, when combined with an appropriate payment mechanism, can effectively meet the demands of controlling medical insurance fund expenditures and hospital management (5, 6). After several years of pilot implementation, China fully rolled out the CHS-DRG (China Healthcare Security-DRG, developed by the National Healthcare Security Administration) payment system in 2021. This system is primarily applied in two major areas: medical insurance payment management and the evaluation of healthcare service performance (7, 8).

As pharmaceuticals represent the primary driver of medical resource consumption in China, the rapid increase in pharmaceutical costs has become a key factor contributing to the unreasonable rise in overall healthcare expenditures. This surge not only escalates the cost of medical services and undermines the healthy functioning of medical insurance funds, but also places a heavier economic burden on patients. Given that public hospitals in China control approximately 90% of healthcare service resources and about 75% of pharmaceutical retail, effectively controlling hospital pharmaceutical costs has become a critical priority in curbing the irrational escalation of medical expenses. In response, the Chinese government has implemented a series of policies aimed at controlling the unreasonable growth of pharmaceutical costs. One of the main objectives of the essential medicine system is to address the issue of rising drug prices driven by profit motives, as well as the shortcomings of the reimbursement mechanism, which have led to an excessive pharmaceutical burden. To tackle the problem of inefficiencies in the distribution of pharmaceuticals, which have resulted in inflated drug prices, China introduced the “Two-invoice System” across all public hospitals in early 2017. Subsequently, in response to hospitals and physicians favoring higher-priced drugs for economic gain, the government also implemented policies to eliminate drug markups in hospitals. However, the strategic pricing behavior of pharmaceutical manufacturers has partly offset the impact of these policies on drug prices, and physicians’ prescribing incentives have not been effectively altered. By 2018, pharmaceutical expenses still accounted for 32.73% of total healthcare expenditure in China, significantly higher than the OECD average of 16.4%. Although surveys indicated that the national volume-based procurement program launched in 2019 substantially reduced the prices of awarded drugs, significant regional disparities still persisted in terms of overall pharmaceutical cost control (9). By 2020, pharmaceutical costs still accounted for more than 30% of total healthcare expenditure in China.

Physicians’ medical practices are critical determinants of changes in healthcare service costs (10), with prescribing behavior being influenced by both direct factors, such as healthcare policies, medical insurance systems, and management strategies, as well as indirect factors like clinical guidelines, prescription restrictions, pharmaceutical sales representatives, and continuing medical education (11). In China, when attempting to influence drug-related expenditures and usage in public hospitals, it is essential to first address physicians’ prescribing behavior (12). The DRG payment system focuses on two key dimensions: “clinical processes” and “resource consumption.” In theory, as long as the DRG payment system can effectively constrain physicians’ prescribing behaviors in public hospitals and reduce pharmaceutical costs, it has the potential to alleviate the problem of unreasonable increases in drug expenditures. Therefore, understanding the impact of DRG payment on the prescribing behavior of physicians in public hospitals at different levels is a crucial area of research that warrants further exploration.

This study takes as a case example a healthcare consortium in J City, China, which includes primary, secondary, and tertiary public hospitals. Using a mixed-methods research approach that combines semi-structured interviews with Ordinary Least Squares (OLS) regression analysis, the study explores the impact of the DRG payment system on physicians’ prescribing behavior. The results indicate that the DRG payment system has a significant effect on physicians’ prescribing practices, a finding that remains robust even after conducting a series of sensitivity tests. Furthermore, this influence varies notably across hospitals of different levels, with the DRG payment system demonstrating a more pronounced reduction in pharmaceutical costs for acute ischemic stroke cases with no more than two comorbidities.

This study makes several contributions, which can be summarized in three key aspects. First, although existing research on the impact of China’s DRG payment policy on physicians’ medical behaviors generally agrees that it helps reduce the consumption of medical resources and lowers pharmaceutical costs (13, 14), the samples used in these studies primarily come from early policy pilot regions. These regions, however, have different payment systems compared to the currently implemented CHS-DRG payment policy (15). This study, by using data collected after the nationwide rollout of the CHS-DRG system, offers a broader and more representative analysis. Second, existing studies predominantly focus on pilot programs in tertiary hospitals, and research on the impact of the CHS-DRG policy within healthcare consortiums that include hospitals of various levels is still insufficient (16). This study, by incorporating data from public hospitals at different levels, explores the variations in the impact of DRG payment across hospitals of differing tiers. Third, most existing studies rely on data based on changes in total pharmaceutical revenues at hospitals, without controlling for the effects of factors such as different departments or types of health insurance on changes in pharmaceutical expenditures (17). This study, however, accounts for the influence of different departments and types of health insurance on pharmaceutical cost variations, providing a more accurate understanding of the policy’s effects.

## 2 Data and methods

### 2.1 Data collection

According to the health and family planning statistical bulletin released by the Health Commission of J City, in recent years, both medical and pharmaceutical revenues in J City have grown rapidly. In 2019, medical income and pharmaceutical income increased by 17.59 and 18.11%, respectively, compared to 2018. Public hospitals provided approximately 90% of inpatient services, while primary healthcare institutions accounted for about 6% of the total service volume. Given these circumstances, this study selected a 3 + 2 + 1 healthcare consortium in J City as the research subject, with the aim of systematically exploring the impact of the DRG payment system on physicians’ prescribing behaviors.

The healthcare consortium chosen for this study consists of one tertiary hospital (Z Hospital), one secondary hospital (N Hospital), and three primary-level hospitals (L Hospital, X Hospital, and J Hospital). Within this consortium, a two-way referral system is in place, with N Hospital serving as the central hospital in the network. This study employs a mixed-methods approach, combining face-to-face semi-structured interviews with Ordinary Least Squares (OLS) regression analysis, to conduct an in-depth investigation into the relationship between DRG payment and physicians’ prescribing behaviors.

### 2.2 Qualitative interviews

Based on an analysis of the current state of research and in alignment with the objectives of this study, a qualitative interview outline was developed. Experts from relevant fields, including those from medical centers, were invited to discuss the feasibility of the interview framework. Subsequently, purposive sampling was employed, and semi-structured interviews were conducted. Trained interviewers, who had undergone standardized training, carried out face-to-face interviews with key stakeholders in the healthcare consortium, including the legal representatives of the central hospital, heads of the medical insurance department, heads of the performance department, heads of the medical records department, as well as clinical physicians from the various hospitals within the consortium. After obtaining informed consent from the interviewees, the interviews were recorded in full audio format, and written notes were taken to document additional information. Interviews were terminated once data saturation was reached, and key information was extracted and organized on the same day. The information collected from institutional interviewees included basic demographic details such as gender, age, professional title, and educational background, as well as their understanding of the DRG payment system and perceptions of its impact on physicians’ prescribing behaviors.

### 2.3 Quantitative research

Acute ischemic stroke, one of the leading causes of death and disability among adults in China, accounts for as much as 78 to 84% of all strokes. Among hospitalized patients in neurology departments, the proportion of those suffering from brain infarctions is the highest, with some hospitals reporting rates exceeding 80%. Given that acute ischemic stroke has been included in clinical pathway management, and there is a substantial body of evidence-based medical guidelines for its pharmacological treatment, prescribing behavior for the treatment of acute ischemic stroke is relatively well-defined. As the central hospital within the healthcare consortium, N Hospital’s key clinical department heads are senior staff from the tertiary hospital Z Hospital, which further strengthens the clinical and operational connection between the two institutions. Additionally, N Hospital is responsible for providing medical and technical guidance to the lower-level hospitals within the consortium. As a result, N Hospital plays a significant and representative role in the pharmacological treatment of common internal medicine diseases within the consortium. The quantitative research component of this study will be based on data from N Hospital, specifically focusing on the inpatient costs and personal information of patients diagnosed with acute ischemic stroke. This data will be systematically analyzed to assess the impact of the DRG payment system on physicians’ prescribing behaviors.

This study utilizes data extracted from the information management system of N Hospital, which includes the complete set of case records and inpatient billing details for all 650 patients diagnosed with acute ischemic stroke, both before and after the implementation of the DRG payment system. The data spans from October 1, 2020, to September 30, 2022, and encompasses various patient characteristics, stroke-related attributes, and prescribing behavior-related factors. Specifically, the demographic characteristics include factors such as gender, age, and medical insurance category; stroke-related characteristics include the department in which the patient was treated, as well as the primary and secondary diagnoses; prescribing behavior characteristics include pharmaceutical costs, length of hospital stay, and other relevant clinical indicators. This comprehensive dataset allows for an in-depth analysis of the impact of the DRG payment system on prescribing behaviors and pharmaceutical costs associated with the treatment of acute ischemic stroke.

To ensure the validity of the data, this study excluded certain case records based on the following criteria: First, data from acute ischemic stroke patients with a hospital stay of fewer than 6 days or longer than 15 days were excluded. Patients with stays of less than 6 days may not have completed the full clinical pathway, while those with stays exceeding 15 days may have moved beyond the acute phase of drug treatment, making their pharmaceutical costs non-comparable to those incurred during the acute phase. Second, data from patients whose primary diagnosis was a condition other than acute ischemic stroke were excluded. These patients were primarily treated for conditions unrelated to acute ischemic stroke, and their drug treatment regimens may have significant deviations from those typically prescribed for acute ischemic stroke. Third, data from acute ischemic stroke patients admitted to the intensive care unit (ICU) were excluded. Since the ICU primarily treats critically ill patients, their drug treatment costs differ substantially from those of patients in other departments who are receiving standard pharmacological treatment for acute ischemic stroke, making such cases non-comparable. Finally, data from high-cost cases that met the criteria for special disease single-case negotiations were excluded. After applying these exclusion criteria, the final sample consisted of 494 cases.

In the quantitative research, the dependent variable is pharmaceutical costs. The DRG payment system primarily works by incentivizing hospitals to reduce consumable medical costs, thereby lowering overall healthcare expenses. As a result, it is hypothesized that the DRG payment system may guide physicians to reduce pharmaceutical costs. In other words, when evaluating the relationship between DRG payment and physicians’ prescribing behavior, if DRG payment has an impact on pharmaceutical costs, this would suggest that it also influences prescribing behavior. The independent variable in this analysis is the DRG payment policy. Control variables include factors that affect physicians’ prescribing behavior. Based on insights from the qualitative interviews and drawing from the research by Yip, the control variables encompass the patient’s admission date, gender, age, number of diagnoses, department type, and medical insurance category (1).

### 2.4 Research methodology

First, descriptive statistics were conducted for each variable to gain an understanding of the basic characteristics of the case records of acute ischemic stroke patients. This initial step allows for a comprehensive overview of the data, including key demographic and clinical information. Next, a correlation analysis was performed to explore the relationships between pairs of variables, in order to investigate how they may be interrelated. This step helps identify potential associations and dependencies among the variables of interest. Finally, a multiple linear regression model was established, with Ordinary Least Squares (OLS) estimation used to analyze the data. By estimating the coefficients of the independent variables, the model allows for the evaluation of the impact of the DRG payment policy on physicians’ prescribing behaviors. The model construction follows the standard procedure for multiple regression analysis, which includes assessing the significance of the variables, as well as the direction and magnitude of their effects on the dependent variable. The specific model structure is outlined as follows:


$$\begin{array}{l} ME_{i}=\beta_{0}+\beta_{1}DRG_{i}+\beta_{2}DA_{i}+\beta_{3}AGE_{i}+\beta_{4}CN_{i}\\ \;+\beta_{5}GEN_{i}+\underset{i}{\sum }ID_{i}+\underset{i}{\sum }MIT_{i}+\varepsilon_{i} \end{array}$$

In this model, ME represents the total pharmaceutical costs incurred during the patient’s hospital stay. DA refers to the date, and this control variable is included to account for the effects of time-related factors that may vary over time but do not change with individual patients. By including DA, the model helps mitigate the potential bias caused by omitted variables that fluctuate over time but remain constant across individuals. AGE denotes the patient’s age. CN refers to the number of comorbidities a patient has, indicating the total count of comorbid conditions present in the patient. GEN indicates the patient’s gender. ID refers to the department in which the patient was hospitalized, providing a categorical distinction between different clinical areas that may influence treatment practices. Lastly, MIT stands for the patient’s medical insurance type, which can have a significant impact on the patient’s treatment plan and pharmaceutical expenditures, as different insurance policies may influence the cost-sharing structure and availability of medications. These variables are included to control for various factors that may affect pharmaceutical costs, thus allowing for a more accurate assessment of the impact of the DRG payment policy on physicians’ prescribing behavior.

To more accurately assess the impact of the DRG payment policy on physicians’ prescribing behavior, the propensity score matching (PSM) method was employed to select the sample. After matching the samples, the model was estimated using Ordinary Least Squares (OLS) regression to evaluate the relationship between DRG payment and physicians’ prescribing behavior. Sensitivity analysis and methods such as variable substitution were then applied to assess the robustness of the regression results. Finally, heterogeneity analysis was conducted on the number of comorbidities, department, and type of health insurance to explore the heterogeneous impact of DRG payment on physician prescribing behavior.

## 3 Results

### 3.1 Qualitative interviews

#### 3.1.1 Demographic characteristics of the interviewed participants

A total of 18 participants completed the interview process for this study. The detailed demographic characteristics of the participants are presented in Supplementary Table 1.

#### 3.1.2 Changes in prescribing behavior

This study focuses on examining the impact of the Diagnosis-Related Group (DRG) payment system on physicians’ prescribing behaviors. Through in-depth interviews, we explored the perspectives and experiences of the participants regarding this issue. The following section presents the findings from the qualitative interviews conducted as part of this research.

**The impact of DRG payment on internal hospital performance evaluation**. In China, public hospitals, in order to ensure their survival, need to integrate the incentive and constraint mechanisms of the healthcare reimbursement policies with the internal performance-based compensation and evaluation systems. According to the results of the interviews, after implementing the DRG (Diagnosis-Related Group) prospective payment model, the hospitals within the healthcare consortium progressively initiated corresponding reforms in their performance-based compensation systems. These reforms included the incorporation of DRG payment-related incentive and constraint mechanisms into their internal performance evaluation frameworks. Specifically, hospitals integrated key DRG-related indicators such as the Case Mix Index (CMI), Relative Weight (RW), time consumption index, cost consumption index, and low-risk mortality rate into their hospital-wide performance assessment systems. This integration aimed to optimize hospital management strategies, assess the medical service capabilities, efficiency, and quality of physicians, and ultimately reduce unnecessary consumption of medical resources.

Although all hospitals within the healthcare consortium have implemented reforms to their performance-based compensation systems in conjunction with the DRG (Diagnosis-Related Group) payment model, the extent to which these reforms have been adopted varies across hospitals of different tiers. This disparity may be related to the distribution of critically ill patients and their choice of healthcare institutions. Primary-level hospitals primarily treat patients with less severe conditions, and the DRG payment rates for cases that involve drug-based treatments tend to exceed the actual medical costs incurred. Consequently, DRG payments at these hospitals are often lower than the costs associated with treating more complex conditions, leading to a situation where the impact of DRG payments on their medical revenue is relatively minor. As a result, primary-level hospitals do not show a strong inclination to adopt DRG-related indicators into their performance-based compensation systems. In contrast, secondary-level hospitals, which treat a broader range of more complex patient cases, frequently encounter situations where medical costs exceed the reimbursement standards set by insurance. These hospitals therefore exhibit a stronger willingness to incorporate DRG-related indicators into their performance evaluation and compensation systems, aiming to reduce excessive medical costs and optimize their medical revenue structures.

In China, critically ill patients primarily seek medical care at tertiary hospitals, particularly those with a Grade A designation, which are equipped to handle the most complex and severe cases. Although these tertiary hospitals receive the highest DRG (Diagnosis-Related Group) payment rates, the wide range of patient conditions they treat leads to considerable variability in medical costs. As a result, tertiary hospitals have become the primary institutions where medical insurance expenditures often exceed the allocated budget, resulting in substantial financial pressures. In response to the financial pressures resulting from medical insurance budget overages, tertiary hospitals have actively implemented internal performance-based compensation reforms in conjunction with DRG payments. This strategy aims to minimize unnecessary medical resource consumption and reduce the economic losses associated with exceeding the limits of medical insurance reimbursement.

**The impact of performance-based compensation reforms on physicians’ prescribing behavior**. In the traditional fee-for-service payment system, physicians’ income is directly linked to the medical costs incurred from the services they provide. This direct financial connection can create an incentive for physicians to recommend unnecessary medical services, potentially leading to excessive testing, over-prescription of medications, and other similar practices. Such behaviors contribute to the irrational escalation of healthcare costs (18). However, the implementation of the DRG (Diagnosis-Related Group) payment model has fundamentally altered the operational management framework of hospitals. The results from the interviews indicate that, under this new system, hospitals have restructured their performance-based compensation evaluations around DRG-related indicators, breaking down established goals and responsibilities and passing them down to clinical physicians at various levels. Physicians become more conscious of both treatment outcomes and cost control in their decision-making processes, ultimately guiding their prescribing behaviors in a more efficient and cost-effective direction. Nonetheless, the impact of performance-based reforms on physicians’ prescribing behavior varies significantly across hospitals of different tiers. The influence is most pronounced among physicians in tertiary hospitals, followed by secondary hospitals, while the effect on primary hospital physicians’ prescribing behavior is less significant.

From the interviews, three primary factors were identified as contributing to the observed impact on physicians’ prescribing behaviors. First, while the performance-based compensation system in hospitals, through the DRG (Diagnosis-Related Group) payment model, encourages physicians to actively manage medication costs by implementing a “surplus retention and reasonable overage sharing” mechanism, primary hospitals face a unique challenge. In these hospitals, the DRG payment rates for drug-based treatment groups are typically higher than the actual costs incurred for treatment. As a result, the performance evaluation system in primary hospitals is unable to effectively influence physicians’ prescribing behavior. In contrast, secondary and tertiary hospitals, which often treat more complex cases, frequently experience medical costs for drug-based treatments that exceed the reimbursement limits set by insurance, particularly in tertiary hospitals. This makes the performance evaluation systems more effective in influencing physicians’ prescribing practices in secondary and tertiary hospitals.

Second, there are significant differences across hospital levels in terms of the availability and range of medications, which directly impacts physicians’ options when prescribing and, in turn, leads to noticeable variations in prescribing behavior depending on the hospital tier. Tertiary hospitals typically have the broadest selection of drug specifications, providing physicians with a wider range of options. To avoid financial penalties resulting from exceeding medical cost limits, physicians in these hospitals tend to select lower-cost medications. Secondary hospitals, while offering fewer drug specifications than tertiary hospitals, still provide physicians with a degree of choice. As a result, the changes in performance evaluations in secondary hospitals do influence physicians’ prescribing behavior, although the effect is less pronounced than in tertiary hospitals. On the other hand, primary hospitals have a more limited selection of medications, leaving physicians with very few options and thus severely restricting their ability to modify prescribing behaviors based on cost considerations.

**The impact of changes in physicians’ prescribing behavior on healthcare quality, treatment innovation, and patient rights**. The results from the interviews reveal that changes in physicians’ prescribing behaviors have multifaceted implications for the healthcare field. In secondary hospitals, these changes have contributed to improving the quality of medical services, while in tertiary hospitals, the alterations in prescribing behavior induced by the DRG (Diagnosis-Related Group) payment model may have a potential inhibitory effect on the innovation of drug treatment technologies, with no significant positive impact on overall hospital medical quality. Additionally, in tertiary hospitals, where critically ill patients are concentrated, the incentive and constraint mechanisms associated with DRG payments may negatively affect the rights of these patients.

Specifically, first, the DRG payment model has encouraged physicians in secondary hospitals to adhere more closely to clinical guidelines when prescribing medications. This shift in prescribing behavior has, in turn, led to an improvement in the overall quality of medical services provided by these hospitals. However, in tertiary hospitals, particularly those with Grade A status, the current diagnostic and treatment protocols, which form the basis of the DRG payment system, somewhat limit the freedom of clinical leaders and specialists in these hospitals to innovate and experiment with new drug treatment regimens. This constraint has had a detrimental effect on the potential for medical innovation and, consequently, on the enhancement of hospital medical quality. Second, critically ill patients who require medication but cannot undergo surgical interventions are primarily concentrated in the emergency departments of tertiary hospitals, particularly those with Grade A designation. In these cases, the DRG payment model has not adequately refined the categorization of critically ill patients who only receive drug treatments. As a result, the weight assigned to the DRG group for these patients tends to be low. Due to the critical condition of these patients, physicians’ prescribing behavior is less influenced by DRG payment, resulting in higher medication costs. As a result, the medical expenses for these patients are almost entirely above the reimbursement standards set by health insurance. In response, hospitals, driven by economic interests, have shifted the burden of costs not covered by insurance to physicians, using performance penalties as a mechanism for transferring these financial responsibilities. In such situations, physicians are faced with a dilemma: they are unable to alter their prescribing behavior due to the critical nature of the patients’ conditions, and they are also reluctant to incur financial losses resulting from exceeding the budgeted insurance reimbursement. This scenario has led to a tendency for tertiary hospitals to avoid accepting or delaying the treatment of critically ill patients, which directly harms patient rights. This issue requires particular attention in the context of changes in prescribing behavior, as it directly relates to patient outcomes and the fairness of healthcare delivery.

#### 3.1.3 Exploration of mechanisms

During semi-structured interviews, it was found that in order to operate smoothly under the DRG payment model, the medical consortium implemented a performance-based compensation reform after introducing DRG payment. This reform integrated the cost control mechanism of DRG payment into the hospital’s performance-based compensation assessment system. To further explore how DRG payment affects physician prescribing behavior, this study obtained the performance-based compensation reform plan of the medical consortium. The new performance-based compensation assessment system, based on hospital culture and combined with the DRG payment model, ultimately influenced physicians’ medical behavior by changing the method of performance compensation calculation. However, due to differences in the functions of hospitals at different levels, the performance compensation reform plans vary across hospitals within the consortium, resulting in differences in the extent to which DRG payment affects physician prescribing behavior.

**Similarities in the performance-based compensation reform plans**. As they are part of the same medical consortium, hospitals at different levels are essentially the same in terms of hospital culture, total performance control, and forms of performance incentives.

First, the performance-based compensation reforms at all levels of hospitals within the medical consortium are based on the same value orientation. The design of the performance system advocates a “knowledge value orientation,” ensuring the principle of “more work, more pay, and better performance, better reward.”

Second, all hospitals in the consortium implement total performance control. According to the Chinese government’s guidance on performance-based compensation reform, hospitals within the consortium reasonably regulate the total performance budget within the approved salary framework, considering the hospital’s financial income and expenditure and cost control capabilities.

Lastly, the forms of performance incentives are the same across hospitals of all levels. Various performance incentives are used in the performance-based compensation reform, including RBRVS performance, DRG performance, cost control performance, quality improvement and incremental performance, and inpatient collaboration performance. RBRVS performance is based on the provincial medical service fee items, excluding drug, consumable, and bed fees, with the remaining medical service items being assessed; DRG performance uses case weights (RW) and case insurance settlement gains and losses as performance assessment indicators, emphasizing the guiding role of knowledge value; cost control performance focuses on controlling departmental controllable costs; quality improvement and incremental performance prioritize high-tech, complex, and critical cases, and high-difficulty surgeries, using service volume as an important assessment target to promote improvements in the hospital’s diagnosis and treatment levels and service capabilities; inpatient collaboration performance assesses the service volume of inpatient medical and nursing staff to enhance medical service capacity.

**Differences in the performance-based compensation reform plans**. The main difference in the performance-based compensation reform plans across hospitals within the consortium lies in the distinct performance compensation calculation rules for hospitals at different levels. Based on job attributes and categories, hospital staff are divided into six categories: medical, nursing, outpatient, medical technology, medical support, and administrative logistics. All hospitals comprehensively use RBRVS and DRG systems to assess performance for medical, nursing, outpatient, medical technology, and medical support units based on the newly developed performance-based compensation plan. The performance calculation for DRG case gains and losses differs by hospital level.

First, in tertiary hospitals, inpatient departments must bear the portion of costs exceeding the DRG payment limit. Cases with multiple comorbidities that cannot undergo surgical treatment are typically concentrated in tertiary hospitals, and the hospitalization costs for these patients often exceed the DRG payment standard. This is a common phenomenon in China, and hospitals must bear the costs beyond the DRG payment limit. However, the higher the hospital’s level, the lower the financial support it receives from the government. The government’s financial contribution to tertiary hospitals is less than 10% of the hospital’s total revenue. Therefore, in the design of the performance-based compensation reform plan, tertiary hospitals transfer the losses exceeding the DRG payment limit to inpatient departments to ensure smooth operation. Physicians, driven by their financial interests, must actively control drug costs. As a result, DRG payment has a greater impact on prescribing behavior in tertiary hospitals. The medication regimens for critically ill patients are more complex, and treating critically ill patients often results in medication costs that cannot be effectively controlled. To minimize personal financial losses, physicians in tertiary hospitals tend to avoid treating critically ill patients. Innovations in drug treatment regimens are also hindered as they often involve costs exceeding the DRG payment standard. The specific performance calculation formula for tertiary hospital reforms is as follows: Monthly performance = RBRVS item points × performance unit price 1 + (DRG case group RW ± case gain/loss) × performance unit price 2 + (quality improvement + increment) × performance unit price 3 + number of inpatient admissions × performance unit price 4 - performance costs ± other assessment project amounts.

Second, in secondary hospitals, inpatient departments can receive rewards if there is a surplus in DRG payment but are not required to bear the costs of overspending. Although the government’s financial support for secondary public hospitals is lower than that for primary hospitals, it is much higher than the financial contribution to tertiary hospitals. This allows secondary hospitals to pass on more benefits to clinical physicians in the performance-based compensation calculation, reducing the pressure on physicians to control drug costs compared to tertiary hospitals. The specific performance calculation formula for secondary hospital reforms is as follows: Monthly performance = RBRVS item points × performance unit price 1 + (DRG case group RW + case balance) × performance unit price 2 + (quality improvement + increment) × performance unit price 3 + number of inpatient admissions × performance unit price 4 - performance costs ± other assessment project amounts.

Lastly, primary hospitals mainly treat patients with relatively mild conditions, and their medical expenses are often well below the DRG payment limit, exhibiting a low multiplier. As a result, the DRG performance component in the performance-based compensation calculation has little to no impact on the prescribing behavior of physicians in primary hospitals.

### 3.2 Quantitative research

#### 3.2.1 Basic information on acute cerebral infarction patient cases

A total of 494 patient samples diagnosed with acute cerebral infarction were collected for this study. Of these, 244 were from the period before the policy implementation, and 250 were from the period after the policy implementation, with the specific details presented in Supplementary Table 2. The average medication cost for these patients was 4,202.30 yuan, with a standard deviation of 1,371.43 yuan, indicating a certain degree of variability in the drug costs within the sample. The mean age of the patients in the sample was 68.63 years, with a standard deviation of 9.69 years, reflecting the age distribution characteristics of the study population. In terms of diagnoses, the average number of comorbidities per patient was 2.59, with a standard deviation of 1.85, suggesting a certain level of variation in the number of comorbidities each patient received. Of the total sample, 244 cases (49.39%) were collected before the policy was enacted, while 250 cases (50.61%) were collected after the policy was implemented. Regarding the gender distribution, there were 305 male patients, accounting for 61.74% of the sample, and 189 female patients, making up 38.26% of the sample.

#### 3.2.2 Correlation analysis

Prior to conducting regression analysis, this study first performed a correlation analysis on the key variables involved. In the correlation matrix, the values below the diagonal represent Pearson’s correlation coefficients, while the values above the diagonal represent Spearman’s rank correlation coefficients. The specific results of the correlation analysis are presented in Supplementary Table 3. The findings from this analysis indicate the following: (1) the correlation between the DRG payment policy and drug costs reached a level of statistical significance, providing a solid foundation for the subsequent regression analysis. (2) The Pearson correlation coefficient between the DRG payment policy and drug costs was −0.181, and the Spearman correlation coefficient was −0.209. Both of these values were statistically significant at the 1% level, indicating a significant negative correlation between the DRG payment policy and drug costs. (3) The correlation coefficients between other independent variables were all below 0.7, suggesting that there are no severe multicollinearity issues between the explanatory variables and the control variables.

#### 3.2.3 Regression analysis

This study initially employed both Ordinary Least Squares (OLS) and Propensity Score Matching with OLS (PSM-OLS) methods to investigate the impact of the DRG (Diagnosis-Related Group) payment policy on physicians’ prescribing behavior. The results of the analyses are presented in Supplementary Table 4.

In the linear regression analysis, control variables including date, age, gender, and the number of comorbidities were incorporated, as shown in Supplementary Table 4. The regression coefficient for the DRG payment policy was −0.146, which was statistically significant at the 1% level. The regression coefficient for the number of comorbidities was 0.172, also significant at the 1% level. However, no significant correlation was found between age and drug costs. These findings suggest that the implementation of the DRG payment policy led to a reduction in drug costs, and that drug costs tend to increase with the number of comorbidities a patient receives. However, age did not appear to have a substantial impact on drug costs. Therefore, based on these results, it can be concluded that the DRG payment policy has a significant impact on physicians’ prescribing behavior.

To enhance the robustness of the estimated results, the study adjusted for the independent variables by introducing an interaction term, DRG * Date, and re-executed the regression analysis. The results, shown in column (2) of Supplementary Table 4, indicate that the coefficient for the interaction term between the DRG payment policy and date was −0.023, significant at the 1% level. The coefficient for the number of comorbidities remained at 0.172, still significant at the 1% level, and the effect of age remained insignificant. These findings further confirm that the implementation of the DRG payment policy consistently results in a significant reduction in drug costs, while maintaining a positive relationship between drug costs and the number of comorbidities. The conclusions are consistent with the results in column (1) of Supplementary Table 4.

To minimize the dependency on model specifications, the study also employed the propensity score matching (PSM) method. The variables—date, age, gender, and the number of comorbidities—were standardized, and the corresponding propensity scores were calculated. Subsequently, a 1:4 caliper matching technique was applied to the matched samples, and regression analysis was performed on these samples. The results are presented in columns (3) and (4) of Supplementary Table 4, respectively. Column (3) presents the results based on the sample within the common support range, while column (4) presents the results based on the successfully matched samples. The conclusions drawn are consistent with those of columns (1) and (2). To enhance the robustness of the estimation results, this study further adjusted the independent variables by introducing the interaction term DRG*Date and re-executed the regression analysis. The results are shown in columns (5) and (6) of Supplementary Table 4, respectively. Column (5) is based on the sample within the common support range, while column (6) is based on the successfully matched samples. The findings are also consistent with those in columns (1) and (2).

#### 3.2.4 Robustness test

**Sensitivity analysis**. To mitigate potential selection bias introduced by the exclusion criteria of inpatient stays shorter than 6 days or longer than 15 days, this study adjusted the design by modifying the exclusion criteria to inpatient stays shorter than 4 days or longer than 14 days. A total of 506 samples were obtained, and the regression analysis was re-conducted. The results did not exhibit significant changes, indicating that the original conclusions are robust and not influenced by selection bias. The detailed analysis results are presented in Supplementary Table 5.

**Substitution of dependent variable**. To validate the robustness of the regression analysis results presented earlier, this study conducted further testing by substituting the dependent variable. Drawing on the methodology used in Yi, this study selected the average daily drug cost as the new dependent variable. The variables, including daily drug cost, date, age, and number of comorbidities, were logarithmically transformed to ensure consistency and accuracy in the analysis (19). After substituting these variables, the results obtained were found to be largely consistent with those presented in Supplementary Table 4. This further reinforces the robustness of the conclusions drawn from the earlier regression analyses, confirming that the findings hold under different model specifications. The results of this analysis are presented in Supplementary Table 6.

**Exclusion of other potential explanations**. To enhance the robustness of the regression analysis results, this study excluded the impact of comorbidity count and the use of low-priced medications on changes in drug costs following the implementation of DRG payments.

First, the regression analysis between DRG and drug costs reveals a significant positive correlation between drug costs and the number of comorbidities. To rule out the possibility that the reduction in drug costs is attributable to a decrease in the number of comorbidities, this study further conducted a regression analysis on the relationship between DRG and comorbidity number. The results indicate a slight increase in the number of comorbidities following the implementation of DRG payments, suggesting that the decline in drug costs is unrelated to changes in comorbidity count. To enhance the robustness of the regression analysis, this study adjusted the independent variables by introducing an interaction term, DRG*date, and re-executed the regression analysis, yielding consistent results. This may be attributed to physicians’ increased focus on accurate disease diagnosis documentation in order to raise case weights. The detailed analysis results are presented in Supplementary Table 7.

Furthermore, some studies suggest that the centralized bulk purchasing policy for pharmaceuticals implemented in China has generally led to a reduction in drug prices, and this has been identified as a key factor contributing to the decrease in drug costs (9). As such, it was essential for this study to investigate whether the centralized bulk purchasing policy applied to drugs commonly used in the treatment of acute cerebral infarction played a role in influencing the observed decline in drug costs. To begin, the study identified the drugs typically used for the treatment of acute cerebral infarction by referencing clinical treatment guidelines, clinical pathways, and consulting with experts in the field. The results of this investigation indicated that, during the acute phase of the disease, the primary medications used were antiplatelet agents and neuroprotective drugs. Subsequently, the study gathered information on the procurement of relevant medications from the Department of Pharmacy at N Hospital. This information focused on the specific drugs included in the national centralized bulk purchasing catalog and their procurement timelines. Supplementary Table 8 presents the details of the drugs from the national bulk purchasing catalog that are used in the treatment of acute cerebral infarction, including the specific drug types, as well as the execution batches and the corresponding procurement dates. The results of the investigation revealed that the centralized bulk purchasing of drugs used in the treatment of acute cerebral infarction did not fall within the time window of the sample selection for this study. This finding ensures that the observed decline in drug costs is not influenced by the timing or implementation of the bulk purchasing policy, thereby reinforcing the validity of the study’s conclusions regarding other potential determinants.

#### 3.2.5 Heterogeneity analysis

**Comorbidity number**. In fact, given that physicians’ prescribing behaviors may be influenced to varying degrees by the DRG payment policy in cases with different comorbidity counts, this could lead to discrepancies in the policy effects reflected in drug costs across different patient groups. Therefore, this study divides the overall sample into two subsamples based on the median comorbidity count, and subsequently conducts regression analysis using model for each of these subsamples.

The regression analysis results indicate that for the group of acute cerebral infarction patients with 0–2 comorbidities, the DRG payment policy has a significant impact on drug costs, with this effect being statistically significant at the 1% level, manifesting as a notable reduction in drug costs. Furthermore, when the independent variable is adjusted to include the interaction term of DRG and date, and the regression analysis is rerun, the results remain consistent with the baseline regression analysis. Additionally, in the group of acute cerebral infarction patients with more than two comorbidities, the effect of the DRG payment policy on drug costs is significant at the 10% level. Even after adjusting the independent variables to include the interaction term of DRG and date, this conclusion still holds. However, in the sample of patients with more than two comorbidities, the impact of the DRG payment policy is less pronounced. This finding may be attributed to the fact that patients with more than two comorbidities typically have more severe conditions, and physicians are more focused on addressing the patients’ medical needs rather than economic considerations, resulting in a smaller change in prescribing behavior compared to cases with fewer comorbidities. This is consistent with the results of the qualitative interviews. Detailed analysis results are presented in Supplementary Table 9.

**Hospital department**. In China, each inpatient is managed by a medical team rather than a single physician, with the team typically consisting of doctors from the same department but at different levels of seniority. As such, the specific department may influence the prescribing behaviors of physicians, which could lead to variations in the policy effects on drug costs across different departments. Therefore, this study conducts regression analysis on the changes in drug costs before and after the implementation of DRG payments across various departments. The 494 sample cases are sourced from the emergency department, rehabilitation medicine department, and internal medicine departments (with internal medicine being further divided into two subcategories: internal medicine I and internal medicine II). As internal medicine I and II belong to the same overarching internal medicine department, the study divides the overall sample into three subsamples: emergency department, rehabilitation medicine department, and internal medicine department. Subsequently, regression analysis using model was performed on each of these three subsamples. The analysis results indicate that prescribing behaviors across all departments are significantly influenced by the DRG payment policy. Detailed analysis results are presented in Supplementary Table 10.

**Type of health insurance**. In China, there is a significant difference in the reimbursement rates between employee medical insurance and resident medical insurance, which may substantially influence patients’ healthcare choices and expenses. As a result, physicians’ prescribing behaviors in cases with different types of insurance may be affected to varying degrees by the DRG payment policy, potentially leading to differences in the policy effects on drug costs across different insurance types. Therefore, this study conducts regression analysis on the changes in drug costs before and after the implementation of DRG payments for cases with different types of health insurance. Among the 494 samples, eight cases were self-paying patients who did not have health insurance, making it impossible to include them in the regression analysis. Consequently, this study performs regression analysis only on the subsamples of 437 patients covered by resident medical insurance and 49 patients covered by employee medical insurance. The analysis results show that, after the implementation of DRG payments, drug costs significantly decreased for both insurance types. Detailed analysis results are presented in Supplementary Table 11.

## 4 Discussion

This study provides insights into how DRG payments affect the prescribing behaviors of physicians in public hospitals at different levels in China, with a particular focus on the underlying influences and an exploration of the mechanisms driving these effects.

### 4.1 The impact of DRG payment on physician prescribing behavior

One of the key objectives of China’s DRG reform is to optimize resource allocation through the standardization of payment rates. In other words, the goal is not just to control total medical costs but to focus on controlling the average per-case drug costs. Previous studies have shown that DRG payments, by setting payment caps, encourage more effective management of pharmaceutical expenditures (20, 21). The findings of this study confirm that DRG payments significantly influence physician prescribing behaviors, and this effect remains robust even after conducting a series of sensitivity checks. This conclusion aligns with the results of several prior studies (13, 22).

However, This influence demonstrates clear variability across hospitals of different levels, with DRG payments having a more pronounced effect on reducing drug costs in cases of acute cerebral infarction with no more than two comorbidities. Physicians’ prescribing behaviors in tertiary hospitals are most significantly affected by DRG payments, followed by secondary hospitals, while prescribing behaviors in primary hospitals are almost unaffected. These findings suggest that the extent to which physicians’ prescribing behaviors are influenced by DRG payments may be closely related to the significant differences in the range of available pharmaceutical specifications across hospitals of varying levels and the treatment complexity of patients’ conditions.

### 4.2 The challenge of balancing costs with patient care outcomes

There are also certain differences in the underlying influences across hospitals of different levels. The DRG reform in China is primarily aimed at effectively controlling irrational cost growth while ensuring the quality of medical services (23). However, the study results suggest that, due to the economic pressure that DRG payment methods may impose on tertiary hospitals when treating high-cost, critically ill patients, some physicians may be inclined to defer such patients in order to avoid the risk of budget overruns. This not only affects the timely treatment of patients but also undermines the equity and accessibility of healthcare services, a finding that has been corroborated by existing research. Cao et al. using a difference-in-differences (DID) approach to analyze DRG payment cases, found that after the implementation of DRG, Chinese public hospitals tended to prioritize lighter cases to optimize economic returns (24).

The underlying causes of this phenomenon may primarily be twofold: First, tertiary hospitals must balance cost control with the provision of high-quality medical services. However, an excessive focus on cost containment may compromise both the quality of care and patient safety. Second, there is an issue of unequal resource allocation across hospitals of different levels, leading tertiary hospitals to bear a disproportionate burden of complex and critically ill patients, which further exacerbates their economic strain.

### 4.3 Inhibit medical innovation in tertiary hospitals

While the research observed that DRG payments promoted rational drug use and improved service quality in secondary hospitals, the implementation of DRG payments in tertiary hospitals might hinder innovation in pharmacological treatment techniques, and could even compromise the rights of critically ill patients. Several studies have corroborated these findings. For example, Zhang et al. observed in their survey-based study that DRG payments reduce over-treatment and improve efficiency but also lead to some negative effects, such as impeding the development of new technologies in tertiary hospitals (25).

The reasons for this phenomenon may primarily be threefold: First, since DRG payment standards are typically based on the cost of existing treatment protocols, hospitals may lack the incentive to adopt new, more costly treatment technologies or medications, which could slow down medical innovation. Second, hospitals may seek to avoid the financial risks associated with new technologies, especially when the long-term effects and cost-effectiveness of these innovations are unclear. Third, in some cases, hospitals may selectively provide innovative treatments to certain patients while excluding others, which may exacerbate inequality in healthcare services.

### 4.4 Influence mechanism

Furthermore, this study delves into the mechanisms through which DRG payments affect physician prescribing behaviors. Previous research has demonstrated that by implementing performance-based management, hospitals can exert a positive influence on the behavior of physicians, particularly in public hospitals (26). Performance management is one of the most critical approaches to hospital administration in China, and it plays an essential role in guiding physicians’ medical practices (17). In 2021, the Ministry of Human Resources and Social Security, along with five other government departments, issued the “Guiding Opinions on Deepening the Reform of the Public Hospital Salary System.” This document clearly emphasized that the reform of public hospital salary systems should be coordinated with reforms in the medical, healthcare, and pharmaceutical sectors, with the ultimate goal of gradually optimizing hospital revenue structures. The results of this study indicate that the active implementation of performance-based salary reforms in Chinese public hospitals, incorporating DRG-related indicators and the corresponding incentive and constraint mechanisms into hospital performance evaluations, is one of the most important factors driving changes in physicians’ prescribing behaviors. Existing studies confirm that after the introduction of DRG payments, hospitals that integrate DRG-related incentive and constraint mechanisms into their performance evaluations can effectively modify the medical behaviors of physicians (25).

### 4.5 Limitations

There were still several limitations. The study did not account for the differences between various types of hospitals and lacked data from private hospitals and specialty hospitals, which may have resulted in sample selection bias.

## 5 Conclusion and recommendations

The conclusions drawn from this study are summarized as follows: First, the research clearly indicates that the DRG payment system has had a significant impact on physician prescribing behavior in China, with this impact exhibiting notable variability across hospitals of different tiers. Specifically, the effect of DRG payments on physicians’ prescribing practices is most pronounced in tertiary medical institutions, followed by secondary hospitals, with a comparatively smaller impact on physicians in primary hospitals. Second, the findings suggest that the shift in prescribing behavior among physicians in secondary hospitals has contributed positively to promoting the practice of rational drug use. However, in tertiary hospitals, DRG payments have not effectively facilitated the standardization of drug treatments. In fact, the implementation of DRG payments may potentially harm the rights of critically ill patients and suppress the innovation of pharmaceutical treatment technologies. Finally, through the analysis of heterogeneity, the study finds that DRG payments have a more pronounced effect on reducing drug costs in cases of acute cerebral infarction with no more than two comorbidities. The reduction effect does not exhibit significant differences across different departments or types of health insurance.

In response to the negative impacts of the DRG payment policy implementation, it is recommended to strike a balance between cost control and the quality of medical services, with particular attention given to its effects on patient outcomes and medical innovation, while maximizing the positive guidance of the DRG policy. First, healthcare insurance departments should continue to refine the medical insurance payment policies. These departments should reasonably adjust DRG payment standards based on factors such as disease types and severity to ensure that hospitals receive adequate compensation when treating critically ill patients. For cases using innovative treatments, higher payment standards or additional subsidies could be established to incentivize hospitals to adopt new technologies. Additionally, based on the specific regional context, it is essential to reasonably adjust the disease group weights for primary hospitals to maximize the positive role of the DRG payment policy in guiding the prescribing behaviors of primary care physicians. Second, hospitals should further strengthen internal management. Hospitals need to optimize their internal performance management systems to ensure effective alignment with the DRG payment policy, thereby guiding physicians in managing drug costs efficiently. Third, the Chinese government should further promote the development of medical consortiums, enabling tertiary hospitals to share resources, complement strengths, and foster collaborative development with secondary and primary hospitals. This would ease the burden on tertiary hospitals and facilitate tiered diagnosis and treatment along with a bidirectional referral mechanism.

## Data Availability

The original contributions presented in the study are included in the article/Supplementary material, further inquiries can be directed to the corresponding author.
