# Supplementary material for: A study on the impact of DRG payment on physicians’ prescribing behavior in China: a case study of a healthcare consortium in J City
Source: Front Public Health. 2025 Apr 28;13:1532622. doi: 10.3389/fpubh.2025.1532622 (PMC12066651; doi:10.3389/fpubh.2025.1532622)
Supplement: Supplementary file 1 [file Table_1.docx]

Supplementary Material

Table 1 Demographic Characteristics of Participants

| **Variable** | **Statistical Results** |
| --- | --- |
| Gender | |
| Male | 14 (77.78) |
| Female | 4 (22.22) |
| Age (Range) | 44 (38-58) |
| Degree | |
| Doctorate | 3 (16.67) |
| Master's Degree | 4 (22.22) |
| Bachelor's Degree | 11 (61.11) |
| Physician's Professional Title | |
| Chief Physician | 1 (7.14) |
| Associate Chief Physician | 8 (57.14) |
| Attending Physician | 5 (35.72) |

Note: Continuous variables are presented as median (range), and categorical variables are presented as proportion (%), n=18.

Table 2 Basic Case Information of Acute Cerebral Infarction Patients

| **Variable** | **Grouping** | **N** | **Mean ± Standard Deviation / %** |
| --- | --- | --- | --- |
| Medication Expenses |  | 494 | 4202.30±1371.43 |
| Age |  | 494 | 68.63±9.69 |
| Comorbidities Number |  | 494 | 2.59±1.85 |
| DRG | | | |
|  | Before DRG | 244 | 49.39 |
|  | After DRG | 250 | 50.61 |
| Gender | | | |
|  | Male | 305 | 61.74 |
|  | Female | 189 | 38.26 |
| Department | | | |
|  | Internal Medicine I | 81 | 16.40 |
|  | Internal Medicine II | 91 | 18.42 |
|  | Emergency Department | 279 | 56.48 |
|  | Rehabilitation Department | 43 | 8.70 |
| Medical Insurance Type | | | |
|  | Resident Health Insurance | 431 | 87.25 |
|  | Employee Health Insurance | 49 | 9.92 |
|  | Non-local Resident Health Insurance | 6 | 1.21 |
|  | Out-of-pocket | 8 | 1.62 |

Note: Continuous variables are presented as mean ± standard deviation, while categorical variables are presented as percentages (%).

Table 3 Correlation Analysis of Key Variables

|  | **ME** | **DRG** | **DA** | **GEN** | **AGE** | **CN** |
| --- | --- | --- | --- | --- | --- | --- |
| ME | 1 | -0.209^***^ | -0.126^***^ | -0.008 | -0.010 | 0.186^***^ |
| DRG | -0.181^***^ | 1 | 0.866^***^ | -0.030 | 0.046 | 0.080^*^ |
| DA | -0.096^**^ | 0.849^***^ | 1 | -0.023 | 0.029 | 0.080^*^ |
| GEN | 0.038 | -0.030 | -0.021 | 1 | 0.092^**^ | -0.203^***^ |
| AGE | 0.005 | 0.049 | 0.039 | 0.102^**^ | 1 | 0.095^**^ |
| CN | 0.264^***^ | 0.086^*^ | 0.096^**^ | -0.171^***^ | 0.101^**^ | 1 |

Note: ^*^ p < 0.1，^**^ p < 0.05，^***^ p < 0.01.

Table 4 Regression Analysis of DRG Payment and Pharmaceutical Costs

| **Variable：ME** | **(1)** | **(2)** | **(3)** | **(4)** | **(5)** | **(6)** |
| --- | --- | --- | --- | --- | --- | --- |
|  | **OLS** | **OLS** | **PSM-OLS** | **PSM-OLS** | **PSM-OLS** | **PSM-OLS** |
| DRG | -0.146^***^ |  | -0.174^***^ | -0.232^***^ |  |  |
|  | (0.035) |  | (0.039) | (0.041) |  |  |
| DRG*DA |  | -0.023^***^ |  |  | -0.027^***^ | -0.037^***^ |
|  |  | (0.006) |  |  | (0.006) | (0.007) |
| DA | 0.023 | 0.022 | 0.023 | 0.045^*^ | 0.023 | 0.044^*^ |
|  | (0.016) | (0.016) | (0.022) | (0.025) | (0.022) | (0.025) |
| AGE | -0.014 | -0.014 | -0.010 | 0.060 | -0.010 | 0.062 |
|  | (0.095) | (0.095) | (0.105) | (0.113) | (0.105) | (0.114) |
| GEN | 0.052^*^ | 0.052^*^ | 0.063^**^ | 0.060^*^ | 0.063^**^ | 0.060^*^ |
|  | (0.029) | (0.029) | (0.030) | (0.032) | (0.030) | (0.032) |
| CN | 0.172^***^ | 0.172^***^ | 0.137^***^ | 0.131^***^ | 0.137^***^ | 0.131^***^ |
|  | (0.032) | (0.032) | (0.034) | (0.036) | (0.034) | (0.036) |
| Constant | 8.103^***^ | 8.109^***^ | 8.129^***^ | 7.753^***^ | 8.131^***^ | 7.746^***^ |
|  | (0.395) | (0.396) | (0.453) | (0.497) | (0.454) | (0.499) |
| *N* | 494 | 494 | 440 | 372 | 440 | 372 |
| adj. *R*^2^ | 0.156 | 0.153 | 0.164 | 0.215 | 0.162 | 0.212 |

Note: The control variables are the same as those in Table 4. The values in parentheses represent White's robust standard errors, ^*^ p < 0.1，^**^ p < 0.05，^***^ p < 0.01. The regression analysis controls for omitted variables that do not change over time but vary by department or health insurance category.

Table 5 Sensitivity analysis

| **Variable：ME** | **(1)** | **(2)** | **(3)** | **(4)** | **(5)** | **(6)** |
| --- | --- | --- | --- | --- | --- | --- |
|  | **OLS** | **OLS** | **PSM-OLS** | **PSM-OLS** | **PSM-OLS** | **PSM-OLS** |
| DRG | -0.167^***^ |  | -0.182^***^ | -0.232^***^ |  |  |
|  | (0.038) |  | (0.038) | (0.040) |  |  |
| DRG*DA |  | -0.026^***^ |  |  | -0.029^***^ | -0.037^***^ |
|  |  | (0.006) |  |  | (0.006) | (0.007) |
| DA | 0.035^*^ | 0.034^*^ | 0.021 | 0.039^*^ | 0.020 | 0.039^*^ |
|  | (0.020) | (0.020) | (0.019) | (0.021) | (0.019) | (0.021) |
| AGE | -0.001 | -0.001 | 0.021 | 0.101 | 0.021 | 0.103 |
|  | (0.098) | (0.098) | (0.103) | (0.110) | (0.104) | (0.110) |
| GEN | 0.050 | 0.049 | 0.060^*^ | 0.063^*^ | 0.060^*^ | 0.063^*^ |
|  | (0.030) | (0.030) | (0.031) | (0.033) | (0.031) | (0.033) |
| CN | 0.147^***^ | 0.147^***^ | 0.107^***^ | 0.109^***^ | 0.107^***^ | 0.109^***^ |
|  | (0.033) | (0.034) | (0.036) | (0.037) | (0.036) | (0.037) |
| Constant | 8.016^***^ | 8.021^***^ | 8.041^***^ | 7.622^***^ | 8.044^***^ | 7.616^***^ |
|  | (0.407) | (0.408) | (0.443) | (0.473) | (0.445) | (0.475) |
| *N* | 506 | 506 | 456 | 387 | 456 | 387 |
| adj. *R*^2^ | 0.144 | 0.142 | 0.160 | 0.217 | 0.158 | 0.214 |

Note: The control variables are the same as those in Table 4. The values in parentheses represent White's robust standard errors, ^*^ p < 0.1，^**^ p < 0.05，^***^ p < 0.01. The regression analysis controls for omitted variables that do not change over time but vary by department or health insurance category.

Table 6 Regression Analysis of DRG Payment and Daily Drug Cost

| **Variable：**  **Daily Drug Cost** | **(1)** | **(2)** | **(3)** | **(4)** | **(5)** | **(6)** |
| --- | --- | --- | --- | --- | --- | --- |
|  | **OLS** | **OLS** | **PSM-OLS** | **PSM-OLS** | **PSM-OLS** | **PSM-OLS** |
| DRG | -0.086^**^ |  | -0.113^***^ | -0.133^***^ |  |  |
|  | (0.038) |  | (0.041) | (0.045) |  |  |
| DRG*DA |  | -0.013^**^ |  |  | -0.017^***^ | -0.021^***^ |
|  |  | (0.006) |  |  | (0.007) | (0.007) |
| Controls | Yes | Yes | Yes | Yes | Yes | Yes |
| Constant | 6.285^***^ | 6.296^***^ | 5.488^***^ | 5.205^***^ | 5.495^***^ | 5.205^***^ |
|  | (0.420) | (0.420) | (0.469) | (0.523) | (0.470) | (0.523) |
| *N* | 494 | 494 | 440 | 372 | 440 | 372 |
| adj. *R*^2^ | 0.160 | 0.159 | 0.176 | 0.171 | 0.174 | 0.169 |

Note: The control variables are the same as those in Table 4. The values in parentheses represent White's robust standard errors, ^*^ p < 0.1，^**^ p < 0.05，^***^ p < 0.01. The regression analysis controls for omitted variables that do not change over time but vary by department or health insurance category.

Table 7 Regression Analysis of DRG Payment and Comorbidities Number

| **Variable：**  **Comorbidities Number** | **(1)** | **(2)** |
| --- | --- | --- |
|  | **OLS** | **OLS** |
| DRG | 0.099^*^ |  |
|  | (0.056) |  |
| DRG*DA |  | 0.017^*^ |
|  |  | (0.009) |
| Controls | Yes | Yes |
| Constant | -0.027 | -0.018 |
|  | (0.641) | (0.642) |
| *N* | 494 | 494 |
| adj. *R*^2^ | 0.226 | 0.226 |

Note: With the exception of the number of comorbidities, the other control variables are consistent with those presented in Table 4. The values in parentheses represent White's robust standard errors, ^*^ p < 0.1. The regression analysis controls for omitted variables that do not change over time but vary by department or health insurance category.

Table 8 Timeline of National Centralized Drug Procurement for Medications Used in the Treatment of Cerebral Infarction

| **Drug Name** | **Centralized Procurement Batch** | **Implementation Date** |
| --- | --- | --- |
| Clopidogrel Tablets | Batch 1 | October 2019 |
| Edaravone Injection | Batch 7 | November 2022 |
| Heparin Injection | Batch 8 | July 2023 |
| Apixaban Injection | Batch 8 | July 2023 |
| Cytidine Diphosphate Choline Injection | Batch 9 | November 6, 2023 (Bidding Date) |

Table 9 Regression Analysis of Subsamples with Different Comorbidities Numbers

| **Variable：ME** | **0-2** | | **>2** | |
| --- | --- | --- | --- | --- |
|  | **(1)** | **(2)** | **(3)** | **(4)** |
|  | **OLS** | **OLS** | **OLS** | **OLS** |
| DRG | -0.161^***^ |  | -0.128^*^ |  |
|  | (0.043) |  | (0.065) |  |
| DRG*DA |  | -0.026^***^ |  | -0.019^*^ |
|  |  | (0.007) |  | (0.010) |
| Controls | Yes | Yes | Yes | Yes |
| Constant | 8.702^***^ | 8.699^***^ | 7.635^***^ | 7.670^***^ |
|  | (0.409) | (0.410) | (0.780) | (0.784) |
| N | 289 | 289 | 205 | 205 |
| adj. R^2^ | 0.114 | 0.113 | 0.186 | 0.183 |

Note: The control variables are the same as those in Table 4. The values in parentheses represent White's robust standard errors, ^*^ p < 0.1，^***^ p < 0.01. The regression analysis controls for omitted variables that do not change over time but vary by department or health insurance category.

Table 10 Regression Analysis of Subsamples with Different Hospital Department

| **Variable：ME** | **emergency department** | | **rehabilitation medicine department** | | **internal medicine department** | |
| --- | --- | --- | --- | --- | --- | --- |
|  | **(1)** | **(2)** | **(1)** | **(2)** | **(1)** | **(2)** |
|  | **OLS** | **OLS** | **OLS** | **OLS** | **OLS** | **OLS** |
| DRG | -0.128^***^ |  | -1.048^***^ |  | -0.134^**^ |  |
|  | (0.036) |  | (0.305) |  | (0.063) |  |
| DRG*DA |  | -0.021^***^ |  | -0.175^***^ |  | -0.020^*^ |
|  |  | (0.006) |  | (0.057) |  | (0.010) |
| Controls | Yes | Yes | Yes | Yes | Yes | Yes |
| Constant | 9.078^***^ | 9.074^***^ | -3.908 | -5.091 | 6.957^***^ | 6.977^***^ |
|  | (0.532) | (0.532) | (3.437) | (4.004) | (1.137) | (1.139) |
| *N* | 279 | 279 | 43 | 43 | 172 | 172 |
| adj. *R*^2^ | 0.058 | 0.059 | 0.101 | 0.076 | 0.127 | 0.123 |

Note: The control variables are the same as those in Table 4. The values in parentheses represent White's robust standard errors, ^*^ p < 0.1，^**^ p < 0.05，^***^ p < 0.01. The regression analysis controls for omitted variables that do not change over time but vary by health insurance category.

Table 11 Regression Analysis of Subsamples with Different Type of Health Insurance

| **Variable：ME** | **resident medical insurance** | | **employee medical insurance** | |
| --- | --- | --- | --- | --- |
|  | **(1)** | **(2)** | **(1)** | **(2)** |
|  | **OLS** | **OLS** | **OLS** | **OLS** |
| DRG | -0.014^***^ |  | -0.029^**^ |  |
|  | (0.004) |  | (0.011) |  |
| DRG*DA |  | -0.002^***^ |  | -0.005^**^ |
|  |  | (0.001) |  | (0.002) |
| Controls | Yes | Yes | Yes | Yes |
| Constant | 2.070^***^ | 2.072^***^ | 2.031^***^ | 2.029^***^ |
|  | (0.030) | (0.030) | (0.065) | (0.066) |
| *N* | 437 | 437 | 49 | 49 |
| adj. *R*^2^ | 0.122 | 0.120 | 0.215 | 0.216 |

Note: The control variables are the same as those in Table 4. The values in parentheses represent White's robust standard errors, ^**^ p < 0.05，^***^ p < 0.01. The regression analysis controls for omitted variables that do not change over time but vary by department.

**Appendix A: Interview Outline for the Study on the Impact of DRG Payment on Physician Prescription Behavior**

1. **Purpose of the Interview**

To understand the current implementation status of DRG payment in public hospitals, the key factors influencing physician prescription behavior, and whether DRG payment has had an impact on physicians' prescribing practices.

1. **Content of the Interview**
2. **Personal Information: Gender, Education, Professional Title, Years of Work Experience**
3. **Hospital's DRG Payment Implementation**
4. Could you explain your understanding of DRG payment? For example, the concept, objectives, roles, and relevant indicators of the policy. What is your opinion on DRG payment?
5. Please describe the basic situation and steps of DRG payment implementation in your hospital.
6. **Effects of DRG Payment Reform**
7. What impact do you think DRG payment has had on the hospital's performance-based compensation assessment? Why do you think these effects occurred?
8. Do you think DRG payment has different impacts on performance-based compensation assessments in hospitals of different levels? Can you explain why？
9. How do you think the performance-based compensation reform driven by DRG payment will affect physicians' prescription behavior? Why?
10. What factors do you think influence physicians' prescription behavior? Are there differences in the impact of performance-based compensation reforms on prescription behavior across hospitals of different levels? Can you explain the reasons for this?
11. After changes in physicians' prescription behavior, what effects do you think this will have on medical quality, patient rights, and the innovation of treatment techniques? Why？

**Appendix B: Main Content of the Semi-Structured Interviews on the Impact of DRG Payment on Physician Prescription Behavior**

| Interview Date | Interviewee | Interviewee ID | Main Content |
| --- | --- | --- | --- |
| 2/12/2023 | N Hospital, Management Staff | N1 | The hospital implemented performance reforms in line with the DRG payment policy, which has been very effective in reducing drug costs and optimizing the hospital's revenue structure. This is beneficial for both the development of the hospital and the patients. |
| 2/12/2023 | N Hospital, Management Staff | N2 | Previously, payments were made based on itemized charges, where patients paid according to the expenses incurred, with no limits on doctors' use of drugs and treatments. With DRG, however, a payment cap is set, and any excess cost results in deductions for the hospital. This encourages doctors to actively control drug costs and reduce medical expenses. |
| 5/12/2023 | N Hospital, Physician | N5 | DRG payment does affect prescription behavior. After the implementation of a cap on medical expenses, drugs that were not necessary according to guidelines, such as nutritional supplements, are no longer prescribed. The standardization of discharge medications has also been regulated, which helps promote rational drug use and benefits medical quality. |
| 8/12/2023 | Z Hospital, Physician | Z1 | Our hospital has already incorporated DRG indicators into performance assessments. The first principle is "more work, more pay," and the second is "better work, better pay." |
| 8/12/2023 | Z Hospital, Physician | Z2 | We treat critically ill patients, but the weight of such cases is not high, often leading to cost overruns. The hospital deducts money from us for the excess. For example, treating diabetic foot or diabetes with pneumonia is very complicated, and medication use is restricted. There have been cases where serious patients are being transferred out to avoid exceeding the budget. If this continues, it will hurt these critically ill patients. |
| 8/12/2023 | Z Hospital, Physician | Z3 | In tertiary hospitals, doctors primarily rely on evidence-based medicine for prescribing. While medical quality has not improved, this system is beneficial for secondary hospitals. When I used to visit your hospital for rounds, I saw that most patients were prescribed multiple boxes of traditional Chinese medicine; now, this has decreased, and there is a greater emphasis on following guidelines. |
| 10/12/2023 | Z Hospital, Physician | Z6 | The impact of DRG payment on doctors' medication practices is significant, especially for us in tertiary hospitals, where the range of drug options is larger. |
| 10/12/2023 | Z Hospital, Physician | Z8 | Our department primarily treats acute and critical cases, most of which involve elderly patients. Many of them do not undergo operative treatments due to their physical condition, so the weight is low. However, drug costs are high, leading to budget overruns, and other departments are unwilling to admit these patients. In some cases, patients are admitted, but due to their condition, they pass away shortly, resulting in low-weight cases. In our hospital, we either face cost overruns or low weight, and the insurance office frequently investigates us. |
| 13/12/2023 | L Hospital, Physician | L1 | DRG payment has little impact on doctors' prescribing behavior. Our clinic mainly treats common and chronic diseases, with payment standards generally high, except for surgical cases. This often leads to low-weight cases. Moreover, our hospital mainly uses essential medicines and centrally procured drugs, so doctors have limited choices. |

Note: The letter in the interview ID represents the code of the interviewee's practicing hospital, while the number indicates the sequence of the interviewee within that hospital.
