# Supplementary material for: A study on the impact of DRG payment on physicians’ prescribing behavior in China: a case study of a healthcare consortium in J City
Source: Front Public Health. 2025 Apr 28;13:1532622. doi: 10.3389/fpubh.2025.1532622 (PMC12066651; doi:10.3389/fpubh.2025.1532622)
Supplement: Supplementary file 2 [file Data_Sheet_1.pdf]

Supplementary Material

Table 1 Demographic Characteristics of Participants

Variable	Statistical Results
Gender	
Male	14 (77.78)
Female	4 (22.22)
Age (Range)	44 (38-58)
Degree	
Doctorate	3 (16.67)
Master's Degree	4 (22.22)
Bachelor's Degree	11 (61.11)
Physician's Professional Title	
Chief Physician	1 (7.14)
Associate Chief Physician	8 (57.14)
Attending Physician	5 (35.72)

Note: Continuous variables are presented as median (range), and categorical variables are presented as proportion (%), n=18.

Table 2 Basic Case Information of Acute Cerebral Infarction Patients

Variable	Grouping	N	Mean ± Standard Deviation / %
Medication Expenses		494	4202.30±1371.43
Age		494	68.63±9.69
Comorbidities Number		494	2.59±1.85
DRG			
	Before DRG	244	49.39
	After DRG	250	50.61
Gender			
	Male	305	61.74
	Female	189	38.26
Department			
	Internal Medicine I	81	16.40
	Internal Medicine II	91	18.42
	Emergency Department	279	56.48
	Rehabilitation Department	43	8.70
Medical Insurance Type			
	Resident Health Insurance	431	87.25
	Employee Health Insurance	49	9.92
	Non-local Resident Health Insurance	6	1.21
	Out-of-pocket	8	1.62

Note: Continuous variables are presented as mean ± standard deviation, while categorical variables are presented as percentages (%).

Table 3 Correlation Analysis of Key Variables

	ME	DRG	DA	GEN	AGE	CN
ME	1	-0.209***	-0.126***	-0.008	-0.010	0.186***
DRG	-0.181***	1	0.866***	-0.030	0.046	0.080*
DA	-0.096**	0.849***	1	-0.023	0.029	0.080*
GEN	0.038	-0.030	-0.021	1	0.092**	-0.203***
AGE	0.005	0.049	0.039	0.102**	1	0.095**
CN	0.264***	0.086*	0.096**	-0.171***	0.101**	1

Note: * $p < 0.1$， ** $p < 0.05$， *** $p < 0.01$.

Table 4 Regression Analysis of DRG Payment and Pharmaceutical Costs

Variable：ME	(1)	(2)	(3)	(4)	(5)	(6)
	OLS	OLS	PSM-OLS	PSM-OLS	PSM-OLS	PSM-OLS
DRG	-0.146***		-0.174***	-0.232***		
	(0.035)		(0.039)	(0.041)		
DRG*DA		-0.023***			-0.027***	-0.037***
		(0.006)			(0.006)	(0.007)
DA	0.023	0.022	0.023	0.045*	0.023	0.044*
	(0.016)	(0.016)	(0.022)	(0.025)	(0.022)	(0.025)
AGE	-0.014	-0.014	-0.010	0.060	-0.010	0.062
	(0.095)	(0.095)	(0.105)	(0.113)	(0.105)	(0.114)
GEN	0.052*	0.052*	0.063**	0.060*	0.063**	0.060*
	(0.029)	(0.029)	(0.030)	(0.032)	(0.030)	(0.032)
CN	0.172***	0.172***	0.137***	0.131***	0.137***	0.131***
	(0.032)	(0.032)	(0.034)	(0.036)	(0.034)	(0.036)
Constant	8.103***	8.109***	8.129***	7.753***	8.131***	7.746***
	(0.395)	(0.396)	(0.453)	(0.497)	(0.454)	(0.499)
N	494	494	440	372	440	372
adj. R^2	0.156	0.153	0.164	0.215	0.162	0.212

Note: The control variables are the same as those in Table 4. The values in parentheses represent White's robust standard errors, * p < 0.1， ** p < 0.05， *** p < 0.01. The regression analysis controls for omitted variables that do not change over time but vary by department or health insurance category.

Table 5 Sensitivity analysis

Variable：ME	(1)	(2)	(3)	(4)	(5)	(6)
	OLS	OLS	PSM-OLS	PSM-OLS	PSM-OLS	PSM-OLS
DRG	-0.167***		-0.182***	-0.232***		
	(0.038)		(0.038)	(0.040)		
DRG*DA		-0.026***			-0.029***	-0.037***
		(0.006)			(0.006)	(0.007)
DA	0.035*	0.034*	0.021	0.039*	0.020	0.039*
	(0.020)	(0.020)	(0.019)	(0.021)	(0.019)	(0.021)
AGE	-0.001	-0.001	0.021	0.101	0.021	0.103
	(0.098)	(0.098)	(0.103)	(0.110)	(0.104)	(0.110)
GEN	0.050	0.049	0.060*	0.063*	0.060*	0.063*
	(0.030)	(0.030)	(0.031)	(0.033)	(0.031)	(0.033)
CN	0.147***	0.147***	0.107***	0.109***	0.107***	0.109***
	(0.033)	(0.034)	(0.036)	(0.037)	(0.036)	(0.037)
Constant	8.016***	8.021***	8.041***	7.622***	8.044***	7.616***
	(0.407)	(0.408)	(0.443)	(0.473)	(0.445)	(0.475)
N	506	506	456	387	456	387
adj. R^2	0.144	0.142	0.160	0.217	0.158	0.214

Note: The control variables are the same as those in Table 4. The values in parentheses represent White's robust standard errors, * p < 0.1， ** p < 0.05， *** p < 0.01. The regression analysis controls for omitted variables that do not change over time but vary by department or health insurance category.

Table 6 Regression Analysis of DRG Payment and Daily Drug Cost

Variable:	(1)	(2)	(3)	(4)	(5)	(6)
Daily Drug Cost	OLS	OLS	PSM-OLS	PSM-OLS	PSM-OLS	PSM-OLS
DRG	-0.086**		-0.113***	-0.133***		
	(0.038)		(0.041)	(0.045)		
DRG*DA		-0.013**			-0.017***	-0.021***
		(0.006)			(0.007)	(0.007)
Controls	Yes	Yes	Yes	Yes	Yes	Yes
Constant	6.285***	6.296***	5.488***	5.205***	5.495***	5.205***
	(0.420)	(0.420)	(0.469)	(0.523)	(0.470)	(0.523)
N	494	494	440	372	440	372
adj. R^2	0.160	0.159	0.176	0.171	0.174	0.169

Note: The control variables are the same as those in Table 4. The values in parentheses represent White's robust standard errors, * $p < 0.1$, ** $p < 0.05$, *** $p < 0.01$. The regression analysis controls for omitted variables that do not change over time but vary by department or health insurance category.

Table 7 Regression Analysis of DRG Payment and Comorbidities Number

**Variable: Comorbidities Number**	**(1)**	**(2)**
	**OLS**	**OLS**
DRG	0.099*	
	(0.056)	
DRG*DA		0.017*
		(0.009)
Controls	Yes	Yes
Constant	-0.027	-0.018
	(0.641)	(0.642)
N	494	494
adj. R^2	0.226	0.226

Note: With the exception of the number of comorbidities, the other control variables are consistent with those presented in Table 4. The values in parentheses represent White's robust standard errors, * $p < 0.1$. The regression analysis controls for omitted variables that do not change over time but vary by department or health insurance category.

Table 8 Timeline of National Centralized Drug Procurement for Medications Used in the Treatment of Cerebral Infarction

**Drug Name**	**Centralized Procurement Batch**	**Implementation Date**
Clopidogrel Tablets	Batch 1	October 2019
Edaravone Injection	Batch 7	November 2022
Heparin Injection	Batch 8	July 2023
Apixaban Injection	Batch 8	July 2023
Cytidine Diphosphate Choline Injection	Batch 9	November 6, 2023 (Bidding Date)

Table 9 Regression Analysis of Subsamples with Different Comorbidities Numbers

Variable：ME	0-2		>2	
	(1)	(2)	(3)	(4)
	OLS	OLS	OLS	OLS
DRG	-0.161***		-0.128*	
	(0.043)		(0.065)	
DRG*DA		-0.026***		-0.019*
		(0.007)		(0.010)
Controls	Yes	Yes	Yes	Yes
Constant	8.702***	8.699***	7.635***	7.670***
	(0.409)	(0.410)	(0.780)	(0.784)
N	289	289	205	205
adj. R^2	0.114	0.113	0.186	0.183

Note: The control variables are the same as those in Table 4. The values in parentheses represent White's robust standard errors, * $p < 0.1$， *** $p < 0.01$. The regression analysis controls for omitted variables that do not change over time but vary by department or health insurance category.

Table 10 Regression Analysis of Subsamples with Different Hospital Department

Variable：ME	emergency department		rehabilitation medicine department		internal medicine department	
	(1)	(2)	(1)	(2)	(1)	(2)
	OLS	OLS	OLS	OLS	OLS	OLS
DRG	-0.128***		-1.048***		-0.134**	
	(0.036)		(0.305)		(0.063)	
DRG*DA		-0.021***		-0.175***		-0.020*
		(0.006)		(0.057)		(0.010)
Controls	Yes	Yes	Yes	Yes	Yes	Yes
Constant	9.078***	9.074***	-3.908	-5.091	6.957***	6.977***
	(0.532)	(0.532)	(3.437)	(4.004)	(1.137)	(1.139)
N	279	279	43	43	172	172
adj. R^2	0.058	0.059	0.101	0.076	0.127	0.123

Note: The control variables are the same as those in Table 4. The values in parentheses represent White's robust standard errors, * $p < 0.1$， ** $p < 0.05$， *** $p < 0.01$. The regression analysis controls for omitted variables that do not change over time but vary by health insurance category.

Table 11 Regression Analysis of Subsamples with Different Type of Health Insurance

**Variable：ME**	**resident medical insurance**		**employee medical insurance**	
	**(1)**	**(2)**	**(1)**	**(2)**
	**OLS**	**OLS**	**OLS**	**OLS**
DRG	-0.014***		-0.029**	
	(0.004)		(0.011)	
DRG*DA		-0.002***		-0.005**
		(0.001)		(0.002)
Controls	Yes	Yes	Yes	Yes
Constant	2.070***	2.072***	2.031***	2.029***
	(0.030)	(0.030)	(0.065)	(0.066)
N	437	437	49	49
adj. R^2	0.122	0.120	0.215	0.216

Note: The control variables are the same as those in Table 4. The values in parentheses represent White's robust standard errors, ** $p < 0.05$， *** $p < 0.01$. The regression analysis controls for omitted variables that do not change over time but vary by department.

Appendix A: Interview Outline for the Study on the Impact of DRG Payment on Physician Prescription Behavior

1 Purpose of the Interview

To understand the current implementation status of DRG payment in public hospitals, the key factors influencing physician prescription behavior, and whether DRG payment has had an impact on physicians' prescribing practices.

2 Content of the Interview

2.1 Personal Information: Gender, Education, Professional Title, Years of Work Experience

2.2 Hospital's DRG Payment Implementation

① Could you explain your understanding of DRG payment? For example, the concept, objectives, roles, and relevant indicators of the policy. What is your opinion on DRG payment?

② Please describe the basic situation and steps of DRG payment implementation in your hospital.

2.3 Effects of DRG Payment Reform

① What impact do you think DRG payment has had on the hospital's performance-based compensation assessment? Why do you think these effects occurred?

② Do you think DRG payment has different impacts on performance-based compensation assessments in hospitals of different levels? Can you explain why？

③ How do you think the performance-based compensation reform driven by DRG payment will affect physicians' prescription behavior? Why?

④ What factors do you think influence physicians' prescription behavior? Are there differences in the impact of performance-based compensation reforms on prescription behavior across hospitals of different levels? Can you explain the reasons for this?

⑤ After changes in physicians' prescription behavior, what effects do you think this will have on medical quality, patient rights, and the innovation of treatment techniques? Why？

Appendix B: Main Content of the Semi-Structured Interviews on the Impact of DRG Payment on Physician Prescription Behavior

Interview Date	Interviewee	Interviewee ID	Main Content
2/12/2023	N Hospital, Management Staff	N1	The hospital implemented performance reforms in line with the DRG payment policy, which has been very effective in reducing drug costs and optimizing the hospital's revenue structure. This is beneficial for both the development of the hospital and the patients.
2/12/2023	N Hospital, Management Staff	N2	Previously, payments were made based on itemized charges, where patients paid according to the expenses incurred, with no limits on doctors' use of drugs and treatments. With DRG, however, a payment cap is set, and any excess cost results in deductions for the hospital. This encourages doctors to actively control drug costs and reduce medical expenses.
5/12/2023	N Hospital, Physician	N5	DRG payment does affect prescription behavior. After the implementation of a cap on medical expenses, drugs that were not necessary according to guidelines, such as nutritional supplements, are no longer prescribed. The standardization of discharge medications has also been regulated, which helps promote rational drug use and benefits medical quality.
8/12/2023	Z Hospital, Physician	Z1	Our hospital has already incorporated DRG indicators into performance assessments. The first principle is "more work, more pay," and the second is "better work, better pay."
8/12/2023	Z Hospital, Physician	Z2	We treat critically ill patients, but the weight of such cases is not high, often leading to cost overruns. The hospital deducts money from us for the excess. For example, treating diabetic foot or diabetes with pneumonia is very complicated, and medication use is restricted. There have been cases where serious patients are being transferred out to avoid exceeding the budget. If this continues, it will hurt these critically ill patients.
8/12/2023	Z Hospital, Physician	Z3	In tertiary hospitals, doctors primarily rely on evidence-based medicine for prescribing. While medical quality has not improved, this system is beneficial for secondary hospitals. When I used to visit your hospital for rounds, I saw that most patients were prescribed multiple boxes of

			traditional Chinese medicine; now, this has decreased, and there is a greater emphasis on following guidelines.
10/12/2023	Z Hospital, Physician	Z6	The impact of DRG payment on doctors' medication practices is significant, especially for us in tertiary hospitals, where the range of drug options is larger.
10/12/2023	Z Hospital, Physician	Z8	Our department primarily treats acute and critical cases, most of which involve elderly patients. Many of them do not undergo operative treatments due to their physical condition, so the weight is low. However, drug costs are high, leading to budget overruns, and other departments are unwilling to admit these patients. In some cases, patients are admitted, but due to their condition, they pass away shortly, resulting in low-weight cases. In our hospital, we either face cost overruns or low weight, and the insurance office frequently investigates us.
13/12/2023	L Hospital, Physician	L1	DRG payment has little impact on doctors' prescribing behavior. Our clinic mainly treats common and chronic diseases, with payment standards generally high, except for surgical cases. This often leads to low-weight cases. Moreover, our hospital mainly uses essential medicines and centrally procured drugs, so doctors have limited choices.

Note: The letter in the interview ID represents the code of the interviewee's practicing hospital, while the number indicates the sequence of the interviewee within that hospital.
